# All-dielectric polarization-sensitive metasurface for terahertz polarimetric imaging

**DOI:** 10.1038/s41598-024-58297-z

**Published:** 2024-03-30

**Authors:** Juhoon Baek, Jaehoon Kim, Jae Hun Seol, Minkyung Kim

**Affiliations:** School of Mechanical Engineering, Gwangju Institute of Science and Technology (GIST), Gwangju, 61005 Republic of Korea

**Keywords:** Absorber, Wavelength converter, Polarization dependence, Photothermal effect, Optics and photonics, Applied optics, Optical materials and structures

## Abstract

Terahertz polarimetric imaging, capable of capturing not only intensity profiles but also the polarization states of the incident pattern, is an essential technique with promising applications such as security scans and medical diagnoses. Recently, a novel approach for terahertz imaging has been proposed using a metasurface absorber that converts terahertz light into a temperature profile. However, polarization remains indistinguishable in the imaging process due to the isotropic geometry of the metasurface. To address this issue, this study introduces an all-dielectric, polarization-sensitive metasurface absorber and showcases its suitability for terahertz polarimetric imaging. Optical and thermal simulations confirm that the polarization dependence of our metasurface is translated into the thermal domain, allowing us to distinguish both intensity and polarization states in the incoming image. Additionally, we demonstrate that polarimetric imaging under general, elliptical polarization is attainable. This metasurface facilitates terahertz polarimetric imaging, eliminating the need for complex setups or bulky components, thereby reducing the form factor and enabling widespread use.

## Introduction

Metasurfaces are subwavelength antenna arrays meticulously designed to exhibit remarkable optical responses^1–8^. Since the interaction between subwavelength antenna and light is strongly tied to the materials, geometries, and arrangements of the metasurfaces, optical properties such as amplitude, phase, polarization, and spatial/temporal frequencies of incident light can be modulated by tailoring the geometries of the metasurfaces. One important merit of the metasurfaces is that they can be designed to behave differently under varying polarizations, offering polarization-sensitive functionalities^9^.

In particular, metasurface absorbers that can provide the high absorption in specific wavelength ranges, whether broadband^10–14^ or narrowband^15,16^, in a subwavelength thickness have emerged as promising alternatives of traditional absorbing materials and find applications in photovoltaics^17,18^, hydrogen production^19^, and microfluidic terahertz (THz) sensors^20,21^. Common to previous metasurface absorbers are their use of metallic films to minimize transmission. However, metal-based metasurface absorbers suffer from high optical losses, low melting points, and high thermal conductivity. Furthermore, the metal ground acts as a heat transfer channel through conduction, diffusing generated heat throughout the metasurface, giving rise to a loss of incoming spatial information. Consequently, there has been a pressing demand for metasurface absorbers exclusively made of dielectric materials^22^.

**Figure 1:**
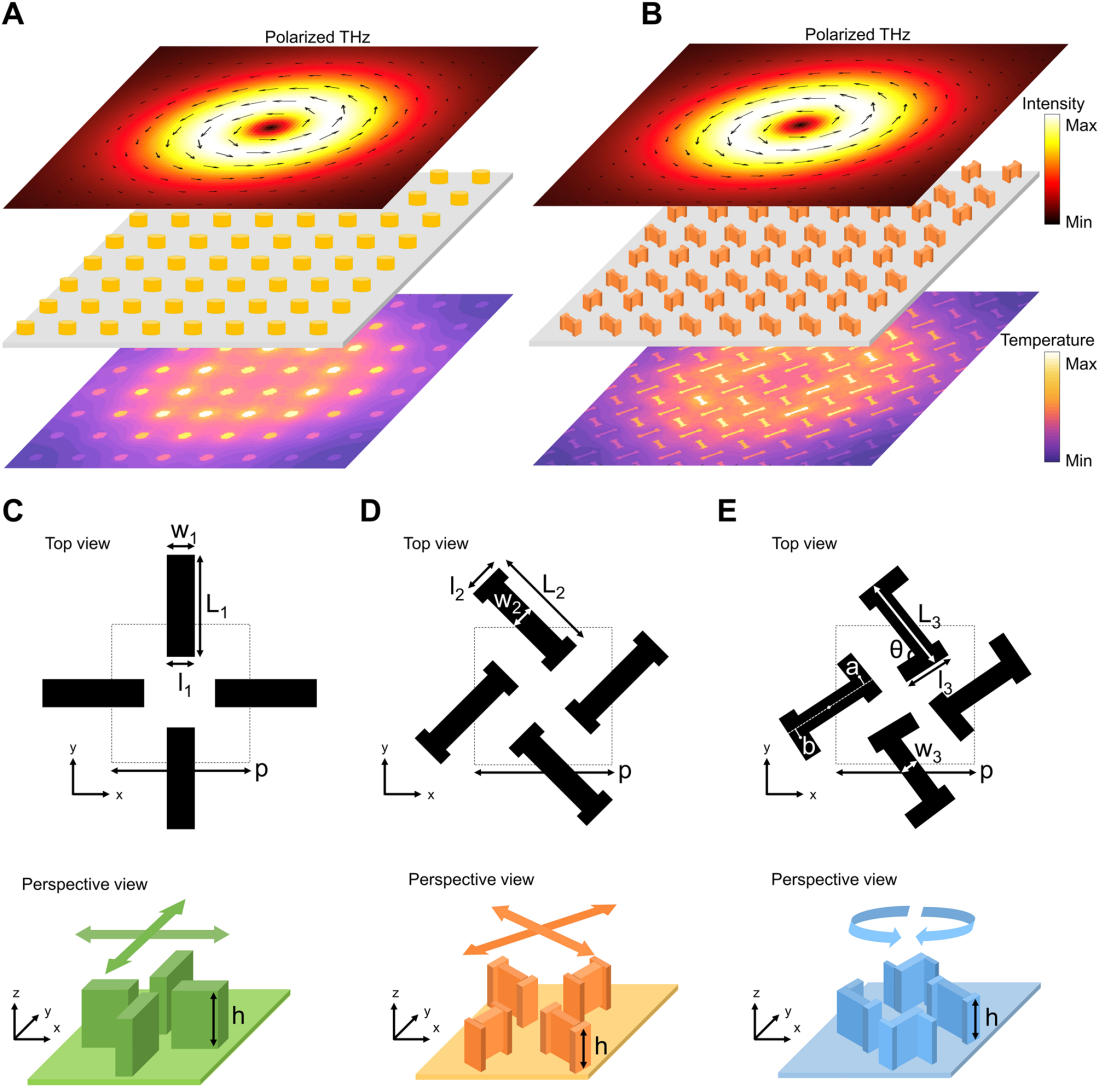
All-dielectric metasurface absorber for THz imaging. (**A**) Metasurface as a wavelength converter for the THz imaging. (**B**) Polarization-sensitive metasurface for the THz polarimetric imaging. (**C**–**E**) Top views of three unit cells and their perspective views as insets for (**C**) horizontally and vertically polarized incidence, (**D**) 45°- and − 45°-polarized incidence, and (**E**) circularly polarized incidence. Parameters labeled *a* and *b* denote distance between the two rods’ center.

One promising application of all-dielectric metasurface absorbers is in THz imaging, where the metasurface serves as a wavelength converter. THz imaging is vital for medical imaging^23,24^, security^25,26^, and material characterization^27^, as it does not involve high-energy radiation, ensuring safety for human subjects, and covers a wide frequency range. However, despite the recent extensive efforts, challenges persist due to the lack of high-power sources and detectors in realizing THz imaging^28^. In contrast, a single-layer, all-dielectric metasurface that absorbs a THz image profile and converts it into an infrared pattern through the photothermal effect offers a novel path to uncooled THz imaging^29,30^. The all-dielectric metasurface, with spatially separated antenna arrays, minimizes in-plane heat spreading, preserving the input image with minimal distortion. The increased temperature profile is then captured in the infrared regime, where the blackbody radiation of room-temperature objects is dominant, by using a conventional infrared camera (Fig. 1A).

While this previous work^27^ demonstrates the usefulness of metasurfaces in capturing intensity profiles of incoming THz light, it lacks polarization information. The isotropic unit structure of the metasurface makes it insensitive to the incident polarization, rendering it unsuitable for polarimetric imaging. To overcome this limitation, we propose an all-dielectric, polarization-sensitive metasurface absorber for THz polarimetric imaging (Fig. 1B). Specifically, three I-shaped resonators are designed to differentiate orthogonal polarizations: (1) horizontal and vertical polarizations, (2) 45° and − 45° polarizations, and (3) right and left circular polarizations (RCP and LCP, respectively). Because of their high geometric anisotropy, these structures exhibit polarization-sensitive absorption characteristics, leading to a spatially inhomogeneous absorption pattern under polarized THz images. For example, tangentially polarized light is strongly absorbed when its polarization direction is parallel to the resonator (Fig. 1B). Because the generated heat spreads out rapidly within the I-shaped resonator but diffuses less to neighboring resonators, the temperature distribution captured by the infrared camera becomes polarization-dependent, enabling THz polarimetric imaging. The absorption characteristics of the metasurface and resulting temperature distribution are obtained by conducting a full-wave optical and thermal simulations, respectively. We confirm that under polarized THz images, the polarization-dependence of our metasurface translates to the thermal domain, allowing us to discern both intensity and polarization states of the incidence. Furthermore, polarimetric imaging is also achievable for general, elliptical polarization by combining two unit cells. This uncooled THz polarimetric imaging enabled by an all-dielectric metasurface absorber holds promise for diverse applications, including medical imaging and security scans.

## Results

### Optical characterization

Three unit cells are devised to distinguish between horizontal and vertical polarizations (Fig. 1C), 45° and − 45° polarizations (Fig. 1D), and circular polarizations (Fig. 1E). These unit cells are periodically arranged on a square lattice, with each unit cell comprising two I-shaped resonators to achieve polarization-dependent absorption. Let us first focus on the unit cell depicted in Fig. 1C. The two resonators within this unit cell are geometrically identical, except that one is rotated by 90° relative to the other. The geometric parameters, such as period (p), length (L and l), width (w), and height (h) of the I-shaped resonators, are determined through particle swarm optimization^31^ to minimize *Q*_*y*_*/Q*_*x*_*A*^*2*^ under horizontally polarized incidence at 1 THz. Here, *Q*_*x*_ and *Q*_*y*_ represent the power absorbed within the x-aligned and y-aligned resonators, respectively, and *A* is the absorption. This indicates that in addition to the high absorption, the x-aligned resonators are optimized to absorb horizontally polarized incidence, while the absorption by the y-aligned resonator is suppressed.

Meanwhile, the I-shaped resonator along the 45° direction is optimized using a similar method (Fig. 1D). The 45°-aligned and − 45°-aligned resonators are optimized to absorb linearly polarized incidence with the polarization parallel to the resonator direction, i.e., 45°- and − 45°-polarized components, respectively. Lastly, to design resonators that are selective to circularly polarized light, three additional parameters *θ*, *a*, and *b* (see Fig. 1E) are introduced. Each resonator is chiral in that it cannot be superimposed onto its mirror image. Consequently, the absorption of each resonator differs under circular polarization. In contrast, the two resonators in the unit cell are mirror images of each other, rendering the entire structure achiral. Therefore, the metasurface exhibits the same absorption under two circularly polarized incidences while displaying resonator-dependent, i.e., geometry-dependent, absorption patterns. The geometric parameters for the three unit cells are summarized in the Supplementary Material Sect. S1. See also Supplementary Material Sect. S5 for the robustness of the metasurface under fabrication errors. Note that the periodicities and heights of the three unit cells are kept identical to ensure compatibility and feasibility. All resonators are constructed from silicon (Si) due to its high refractive index, enabling high polarization sensitivity and absorption. The substrate is selected as an 8 μm-thick polydimethylsiloxane (PDMS) for its low thermal diffusivity and high infrared emissivity. The refractive indices of Si and PDMS are 3.1637 + 0.0948i and 1.3152 + 0.0981i, respectively^30^.

**Figure 2:**
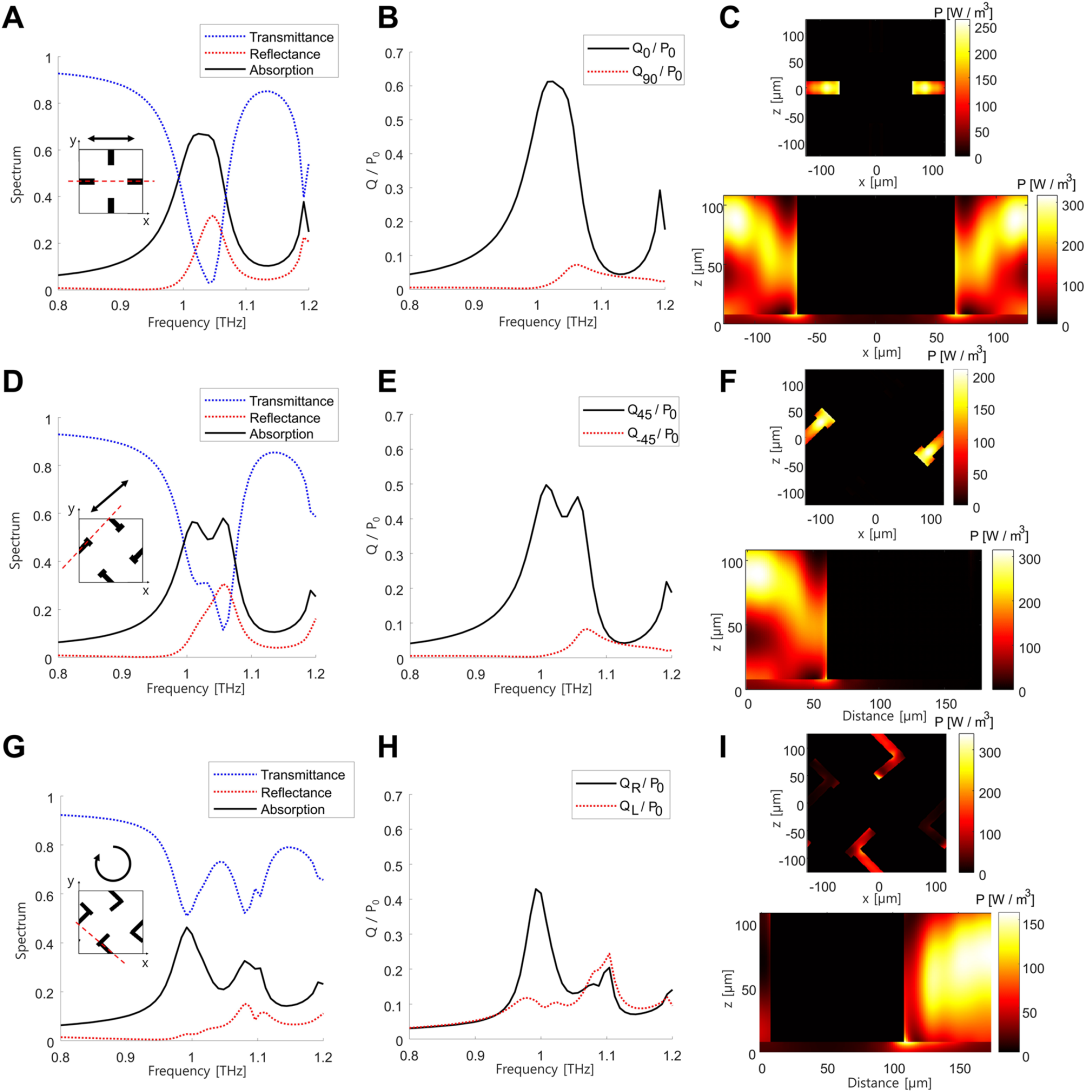
Optical responses of the metasurfaces. Left column shows transmittance (blue), reflectance (red), and absorption (black) spectra. Incident polarization is represented using black arrow in insets. Middle column presents absorbed power spectra in each resonator, and right column shows power dissipation density distribution at 1 THz (top: at the middle of the resonators and bottom: along the red dashed line) under different incident polarizations. (**A**–**C**) correspond to a unit cell designed for selective absorption of horizontally or vertically polarized incidence under horizontally polarized incidence, (**D**–**F**) correspond to a unit cell designed for selective absorption of 45°- or − 45°-polarized incidence under 45°-polarized incidence, and (**G**–**I**) correspond to a unit cell designed for selective absorption of circularly polarized incidence under RCP incidence. Subscripts R and L indicate RCP and LCP, respectively.

The absorption spectra of the three unit cells are simulated using a finite-element method (COMSOL Multiphysics, version: 6.0, https://www.comsol.com/comsol-multiphysics, Fig. 2) and cross-verified with a finite-difference time-domain (FDTD) based commercial software (Lumerical FDTD, version: 1.8.3578, https://www.ansys.com/products/optics/fdtd). The source is incident from air onto the metasurface, with boundaries along the x- and y-axes set to be periodic. The unit cell consisting of the x- and y-aligned resonators exhibits absorption of 0.55 at 1 THz under horizontally polarized incidence (Fig. 2A). This relatively lower absorption, compared to other metasurface absorbers, is attributed to its polarization selectively, because of which only one resonator out of two contributes to the absorption. The absorption is not spectrally broad but the target frequency can be adjusted by tuning geometric parameters (Supplementary Material Sect. S5). For further investigation, we calculate the power dissipation density using the following expression:

1 $$P = \frac{1}{2} {\varepsilon }_{0}{ \varepsilon }_{r}^{"} \omega {\left|E\right|}^{2},$$

where ${\varepsilon }_{0}$ is the permittivity of vacuum, ${\varepsilon }_{r}^{"}$ is the imaginary part of relative permittivity, $\omega$ is the angular frequency, and $E$ represents the electric field. The ratio of power absorbed by a specific resonator is determined by integrating the power dissipation density (Eq. 1) across the resonator’s volume and dividing it by the input power ${P}_{0}$, which is expressed as follows:

2 $$Q=\int PdV/{P}_{0}.$$

Note that $Q$ is a dimensionless quantity and independent of ${P}_{0}$.

Under horizontal polarization, the power absorbed by the x-aligned resonator (black in Fig. 2B) significantly surpasses that absorbed by its orthogonal counterpart (red), with a ratio between the two reaching 94.5 at 1 THz. This unequivocally confirms the selective absorption of horizontally polarized THz radiation by the x-aligned resonator. The cross-sectional distribution of power dissipation density clearly illustrates this polarization-dependent absorption (Fig. 2C). The top and bottom profiles display the density at the middle of the resonator and along the red dashed lines (see Fig. 2A, inset), respectively, and all power densities in Fig. 2 are computed for |E|= 1 V/m. While the power dissipation density in the y-aligned resonator is negligible, the x-aligned resonator exhibits a predominant response due to its dipole-like behavior. This implies that the incident polarized light is mostly absorbed by the x-aligned resonator and subsequently converted into heat. Given that the horizontal and vertical polarizations are four-fold rotational symmetric and so is the metasurface, vertically polarized incidence is primarily absorbed by the y-aligned resonator, with minimal interaction with the x-aligned one.

Similar results are observed in other unit cells. Under 45°-polarized incidence, the unit cell comprising 45° and − 45° aligned resonators demonstrates absorption of approximately 0.52 at 1 THz (Fig. 2D). The decomposition of absorbed power for the two orthogonal resonators reveals a significantly high power contrast between them, reaching 98.3 (Fig. 2E,F). Meanwhile, handedness-dependent absorption is evident in the last unit cell (Fig. 2G–I). Absorption spectra under two opposite circular polarizations, i.e., RCP and LCP, appear degenerate due to the mirror symmetry of the unit cell, with absorption approximately of 0.44 at 1 THz (Fig. 2G). In contrast, the distribution of the power dissipation density demonstrates that each resonator is handedness-sensitive (Fig. 2H,I). Under LCP incidence, the power dissipation density is dominant at the LCP resonator, while it is relatively less pronounced at the RCP resonator. In comparison to the unit cells designed for linear polarizations, the unit cell for circular polarization exhibits relatively lower absorption and less polarization sensitivity. One reason for this lower sensitivity lies in the difficulties in finding all three resonators arranged with the same periodicity. Nevertheless, the absorbed power ratio between the two resonators reaches 4.4, indicating that approximately 82% of the absorbed power is concentrated within one resonator (Fig. 2H), and this can be further enhanced by incorporating chiral absorbers^32,33^ or by first optimizing the unit cell for circular polarization and then repeating the optimization of unit cells for linear polarizations.

### Thermal characterization

**Figure 3:**
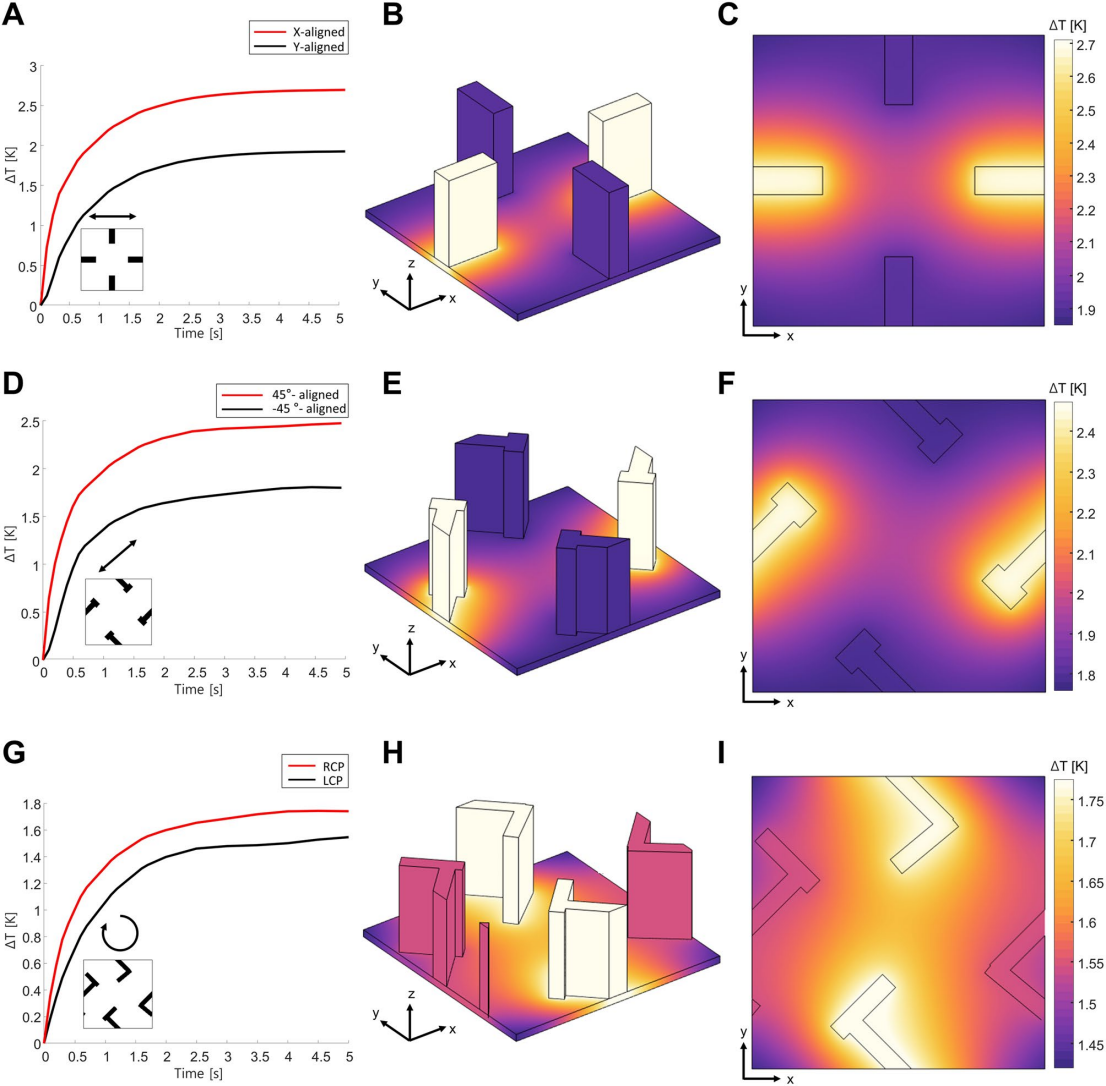
Thermal responses of the metasurface. Left column presents time evolution of the average temperature in each resonator. Incident polarization is indicated as black arrow in insets. Middle and right columns present perspective and top views of the stationary state temperature, respectively. Distributions shown in the right column are at the middle of the substrate.

To validate the polarization-dependent absorption and its consequent effect on temperature variation, heat simulations under both time-transient and stationary conditions are performed using COMSOL Multiphysics (Fig. 3). We conduct a large-scale simulation, utilizing 8 by 8 arrays of each unit structures on a 9*p* by 9*p*-sized substrate (see Supplementary Material Sect. S2), with a Gaussian incidence featuring a beam waist of 400 μm and a total power of 100 μW. This allows us to account for in-plane thermal transport and observe temperature changes at the central unit cell. The power incident on the central unit cell is approximately 23.4 μW, corresponding to 23.4% of the total incident power. The power dissipation density (Eq. 1) obtained from optical simulations (Lumerical FDTD) is used as a volumetric heat source. The absorbed heat in each resonator is dissipated through two channels, i.e., its bottom and top surfaces, in which heat conduction and natural convection dominate, respectively. The heat transfer via the side walls of each resonator is negligible and thus not considered. Furthermore, radiation from the surface to the ambient environment is accounted for at all outer boundaries. The initial and ambient temperatures are both set to room temperature (298 K) (see Supplementary Material Sect. S3 for further details of the thermal analysis, e.g., the thermal properties of the materials and the suitability of the employed thermal boundary conditions).

The time-evolution of the average temperature within each resonator is depicted in the left columns of Fig. 3. Notably, the temperatures within the resonators stabilize to their stationary values after a few seconds, and in both transient and stationary states, resonators with higher absorption exhibits correspondingly higher average temperatures, as anticipated. The response times of three unit cells are 0.83, 0.73, and 0.85 s, respectively (see Supplementary Material Sect. S7 for details on thermal response time). The perspective and top views at the stationary state show that the absorbed power leads to a uniform increase in temperature across the resonator without keeping its power distribution (middle and right columns in Fig. 3, respectively). These uniform temperature distributions within the resonators originate from the short time scale for heat conduction within each resonator. Meanwhile, because of the substantial difference between the conduction thermal resistance inside the resonators and the natural convection/conduction thermal resistance from the resonators toward the environment, the two resonators that selectively interact with the incident polarization exhibit distinct stationary state temperatures.

In other words, heat generated by the absorbed power diffuses rapidly within the resonator, causing its temperature to rise, while it spreads out significantly slower toward the ambient atmosphere or the lower PDMS film. Therefore, the temperature distribution forms uniformly inside the resonators, which reveals the polarization dependence of the optical absorption to the thermal domain. For example, the x-aligned resonator, which absorbs the horizontally polarized incidence, experiences a temperature increase of up to 2.71 K, while the y-aligned resonator only reaches 1.85 K (Fig. 3B). This resonator-dependent temperature variation is also reflected in the temperature profile within the substrate (Fig. 3C). Likewise, the 45°-aligned and RCP resonators exhibit higher temperatures under 45°-polarized and RCP incidence, respectively (Fig. 3E,H). These results affirm that our metasurface unit cells effectively transform the incident polarized image into a temperature distribution, opening a new avenue for THz polarimetric imaging.

### Polarimetric imaging under linearly or circularly polarized incidence

**Figure 4:**
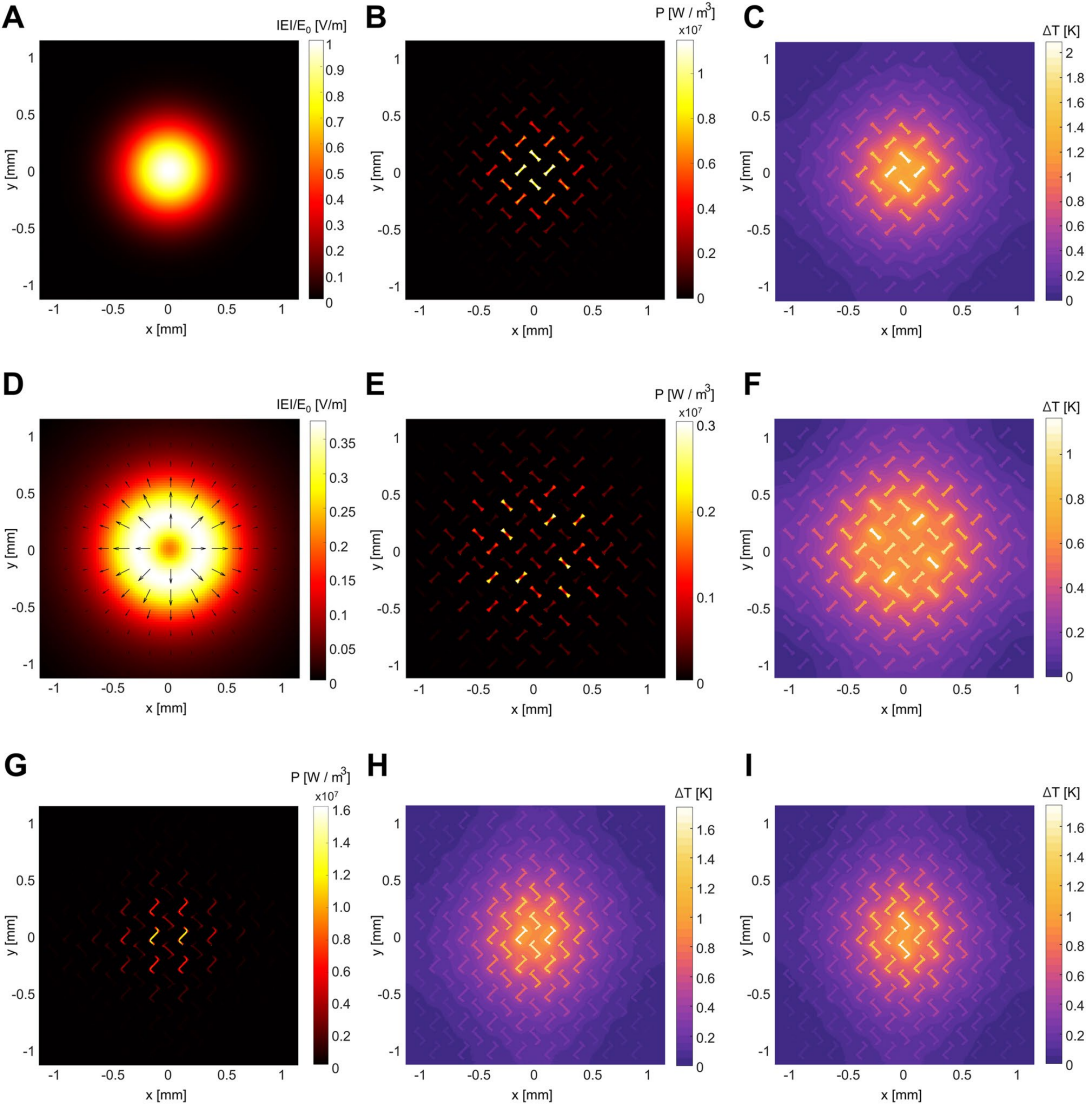
Expected infrared image under patterned illumination. (**A**) Normalized electric field amplitude of the incidence, (**B**) power dissipation density, and (**C**) the resultant infrared image under unpolarized Gaussian. (**D**) Amplitude of the incidence, (**E**) heat dissipation density, and (**F**) the resultant infrared image under radially polarized doughnut-shaped incidence. The incident amplitude is normalized by E_0_ = 548 V/m, the maximum amplitude of the Gaussian shown in (**A**). (**G**) Heat dissipation density and (**H**,**I**) the resultant infrared images under (H) LCP and (**I**) RCP polarized Gaussian. The amplitude distribution of the circularly polarized incidence is same as (**A**). All infrared images show the relative temperature.

To demonstrate polarimetric imaging, we examine the transformation from the incident THz image pattern to a temperature distribution under patterned illumination. Figure 4 depicts power dissipation density and temperature distribution of two metasurfaces, one consisting of 45°- and − 45°-aligned resonators (Fig. 1D) and the other consisting of LCP and RCP resonators (Fig. 1E), both subjected to polarized THz light. Initially, under unpolarized Gaussian incidence (Fig. 4A), the 45°- and − 45°-aligned resonators exhibit equal absorption and thus the power dissipation density reproduces the incident intensity profile (Fig. 4B). The temperature image expected to be captured by the infrared camera also displays a similar pattern (Fig. 4C; see Supplementary Material Sect. S4 for additional details). Under this unpolarized illumination, the temperature rise within the two orthogonal resonators is identical.

We then alter the incidence to a radially polarized doughnut-shaped pattern to demonstrate the metasurface's capability to discern spatial profiles of both intensity and polarization states (Fig. 4D). The power dissipation density depends on both intensity and polarization (Fig. 4E). The overall distribution mirrors the intensity pattern, with resonators aligned with the incident polarization state exhibiting dominant density. Consequently, the resulting temperature profile closely resembles this power dissipation density, with the distinction that the temperature within each resonator remains uniform due to rapid heat diffusion (Fig. 4F). Thus, the temperature profile is affected by both the intensity profile and the resonator geometry, with the latter containing polarization-dependent characteristics. This enables us to identify the spatial distribution of the incident polarization by merely examining the temperature image captured by an infrared camera.

Lastly, the metasurface comprising LCP and RCP resonators under circularly polarized Gaussian incidence is investigated (Fig. 4G–I). The power dissipation density under LCP incidence (Fig. 4G) is reproduced by the temperature profile (Fig. 4H). In contrast, a distinct temperature pattern emerges under RCP incidence (Fig. 4I), indicating that the handedness of the circularly polarized incidence can be distinguished using our metasurface. Note that the spatial resolution of the polarimetric imaging is approximately $\sqrt{2}$ times larger than in the isotropic case, as each unit cell contains two resonators. This resolution corresponds to the periodicity of the metasurface, which is approximately 250 μm. Considering that the temperature resolution of commercially available infrared cameras is typically several orders of magnitude smaller than 1℃, our metasurface can provide the polarimetric imaging in commercial applications using conventional infrared cameras. Finally, while we use the input power of 100 μW in our study, the metasurface works well under other input powers (see Supplementary Material Sect. S6 for details).

### Polarimetric imaging under elliptical polarizations

**Figure 5:**
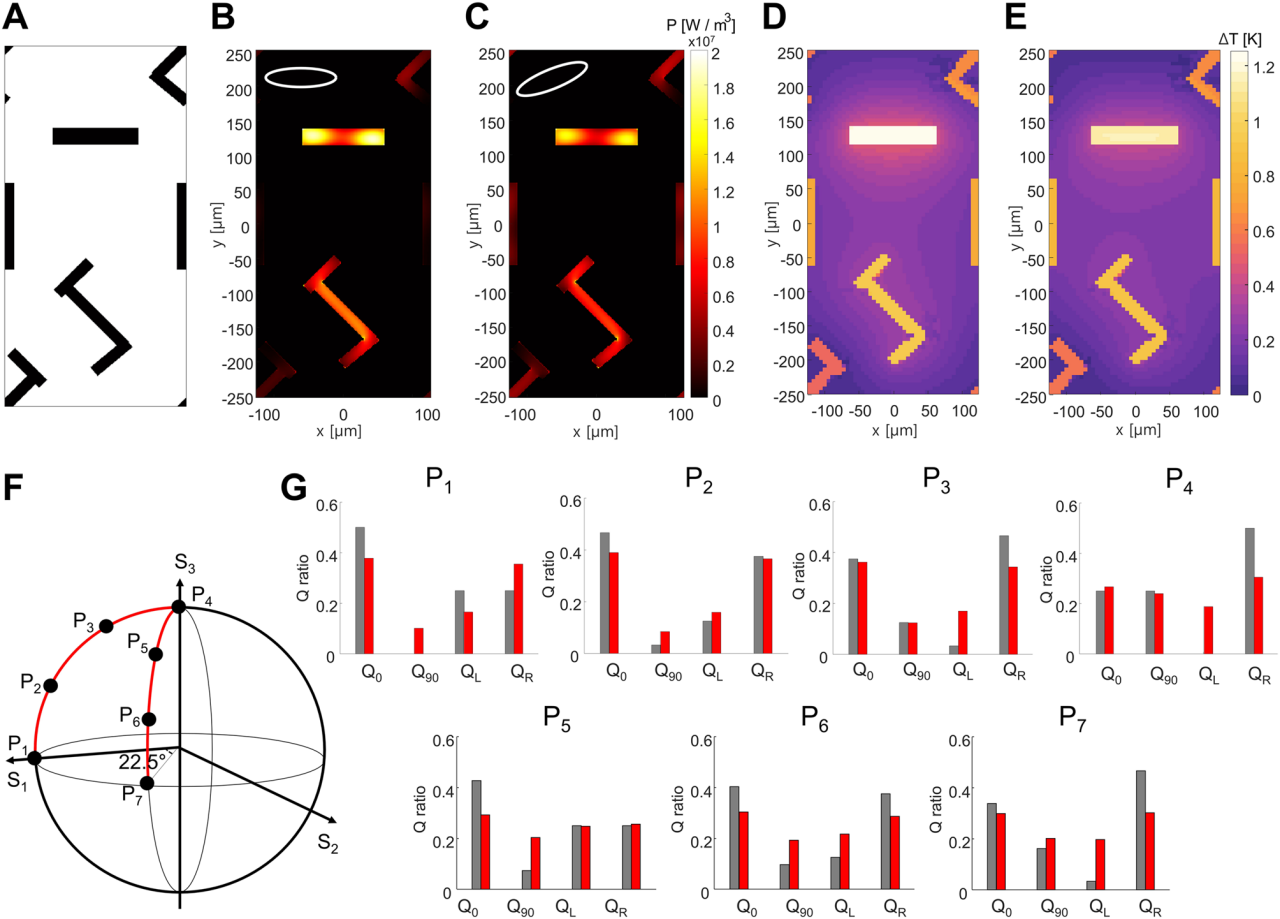
Metasurface supercell structure for elliptically polarized incidence. (**A**) The unit cell of superstructure consisting of four resonators. (**B**,**C**) Power distribution density under two elliptical polarizations, shown as insets, and (**D**,**E**) the resultant temperature at the stationary state. (**F**) Poincaré sphere representing seven different polarizations, P1–P7. (**G**) Ideal (gray) and simulated (red) power ratio absorbed at four resonators.

Finally, to showcase the versatility of our metasurface under various elliptical polarizations, we construct a supercell by combining two unit cells shown in Fig. 1C,E (Fig. 5A). We analyze the power dissipation density and the resulting temperature rise under two distinct incident polarizations (Fig. 5B–E, with polarization states shown in the insets of B and C). Note that a unit cell within this superstructure has twice the periodicity along the y-axis, resulting in a reduction in spatial resolution by half.

Under elliptically polarized incidence with a relatively strong horizontal and RCP component (P2), the x-aligned and RCP resonators absorb strongly (Fig. 5B) and consequently exhibit higher temperatures compared to the y-aligned and LCP resonators (Fig. 5C). Meanwhile, when the incidence corresponds to P6, the power density in the x-aligned resonator decreases, while that of the y-aligned resonator increases due to the stronger vertical component of the incident polarization (Fig. 5D). Therefore, the temperature of the y-aligned resonator increases, while that of the x-aligned resonator decreases (Fig. 5E). This clearly demonstrates that the polarization state of the elliptically polarized incidence is converted into the temperature profile with nice agreement.

The absorbed power ratio in each resonator is quantitatively compared under seven different incident polarizations shown in the Poincaré sphere (Fig. 5F). Gray bars in Fig. 5G denote the power ratio of each polarization component within the incidence obtained by decomposing it into its polarized components, i.e., ${Q}_{i}={\left({P}_{{\text{inc}}}\cdot {P}_{i}\right)}^{2}$ where ${P}_{{\text{inc}}}$ is the Jones vector of the incident polarization and ${P}_{i}$ is the polarization state for which each resonator is designed: ${P}_{0}=\left(1, 0\right), {P}_{90}=\left(0, 1\right), {P}_{L}=\frac{1}{\sqrt{2}}\left(1, i\right), {P}_{R}=\frac{1}{\sqrt{2}}\left(1, -i\right).$ Red bars represent the integrated power ratio in each resonator obtained by numerical simulation and Eqs. (1 and 2) (Fig. 5G). The discrepancy between the ideal and simulated results originates from the incomplete polarization sensitivity, particularly of the LCP and RCP resonators (Fig. 2H), and the coupling between neighboring resonators. The unit cells are designed under the periodic arrangement assumption and the deviation from this periodic condition degrades the overall performance. Nevertheless, these results demonstrate that the supercell structure effectively distinguishes the polarization state of THz images qualitatively, even under general elliptical polarization. Optimization of the unit cell of the superstructure itself, without merging two independently designed unit cells, or using other advanced optimization techniques may further improve polarimetric performance.

## Discussion

In summary, this study proposes an all-dielectric, polarization-sensitive metasurface for THz polarimetric imaging. In contrast to traditional THz imaging methods, where polarization remains indistinguishable, our metasurface transforms the spatial profile of the incident polarization state into a temperature image, which can be readily captured using a standard commercial infrared camera. We present three unit cells designed to differentiate between three distinct sets of orthogonal polarizations, and the choice of a particular unit cell or their combination can be made based on specific requirements. The absorption of our metasurface is approximately half, as each unit cell comprises two distinct resonators, one strongly interacting with the incidence while the other does not. Using current principle, the half absorption seems inevitable, considering the inherent short time scale of heat conduction within the solid resonator. Searching for more efficient metasurfaces by capitalizing on their multifunctionality would be promising.

### Supplementary Information

Supplementary Information.

## Data Availability

The datasets generated during and/or analysed during the current study are available from the corresponding author on reasonable request.
