# Supplementary material for: All-dielectric polarization-sensitive metasurface for terahertz polarimetric imaging
Source: Sci Rep. 2024 Mar 30;14:7544. doi: 10.1038/s41598-024-58297-z (PMC10981697; doi:10.1038/s41598-024-58297-z)
Supplement: Supplementary file 1 — Supplementary Information. [file 41598_2024_58297_MOESM1_ESM.pdf]

Supplementary Material of

All-dielectric polarization-sensitive metasurface for terahertz polarimetric imaging

Juhoon Baek[1+], Jaehoon Kim[1+], Jae Hun Seol[1*], Minkyung Kim[1**]

[1]School of Mechanical Engineering, Gwangju Institute of Science and Technology (GIST), Gwangju 61005, Republic of Korea

+These authors contribute equally to this work

*jhseol@gist.ac.kr

**m.kim@gist.ac.kr

1. Geometric parameters

Resonator	Parameter	Value
X / Y-aligned	L_1	119.9 μm
	l_1	24 μm
	w_1	24 μm
45° / -45°-aligned	L_2	119.2 μm
	l_2	35.1 μm
	w_2	19.8 μm
RCP / LCP	L_3	134 μm
	l_3	63.2 μm
	w_3	15.8 μm
	a	46.0 μm
	b	70.2 μm
	θ	84.73°

Table 1. Geometric parameters.

Period p = 251.1 μm and height h = 100 μm are fixed for all resonators.

2. Metasurface for large-scale simulations

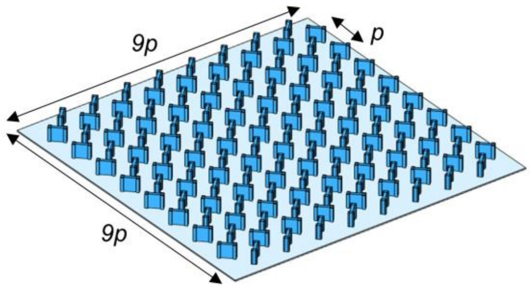

Fig. S1. A schematic diagram of the metasurface used for large-scale simulations.

3. Thermal analysis

The temperature distribution of each individual unit cell in an 8 by 8 arrays, as well as the change in temperature over time, are determined using the stationary equation (Eq. S1) and the time-dependent equation (Eq. S2), respectively. The equations are given as follows:

$$\nabla \cdot \boldsymbol{q} = Q, \quad \text{(S1)}$$

$$\rho C_P \frac{\partial T}{\partial t} + \nabla \cdot \boldsymbol{q} = Q, \quad \text{(S2)}$$

where $\boldsymbol{q}$ is the heat flux, which is expressed in the product of the thermal conductivity of substance and its temperature gradient, *i.e.* $\boldsymbol{q} = -k\nabla T$, Q is the heat source obtained from the power dissipation density derived from the optical simulations, ρ is the density of material, and C_P is the heat capacity. The thermal conductivity, density, and heat capacity of the silicon (Si) resonators, which depend on temperature, are calculated by utilizing the built-in functions of COMSOL Multiphysics. Additionally, the thermal conductivity, density, and heat capacity of polydimethylsiloxane (PDMS) are 0.16 $\mathrm{W/m \cdot K}$, 970 $\mathrm{kg/m^3}$, and 1460 $\mathrm{J/kg \cdot K}$, respectively, which are the standard material properties of PDMS.

All surfaces of the structure, which consists of Si resonators and the PDMS film, are exposed to the external air, and a Gaussian beam is located in the center of the structure. Each resonator conducts the heat generated by the Gaussian beam to the PDMS underneath, and the resonator and the PDMS eventually dissipate the heat to the environment via thermal radiation and natural convection. The thermal radiation emitted from the surfaces of the structure to the surrounding air is denoted as follows:

$$q_{\mathbf{rad}} = \epsilon\sigma(T_{\text{amb}}^4 - T^4). \quad \text{(S3)}$$

Note that $q_{\mathbf{rad}}$ is the thermal radiation heat flux, ϵ is the emissivity, σ is the Stefan-Boltzmann constant, and T_{amb} is the temperature of the external air. The boundaries for natural convection are the upper surfaces of each resonator and the upper/lower surfaces of the PDMS film. Because the peripheral side walls of the PDMS film, which is 8-μm-thick, exhibit a negligible airflow compared to those on the upper or lower surfaces of the PMDS film [1], it is justified not to take the contribution of natural convection on the side walls of the PDMS film into account. Natural convection on the side walls of the 100-μm-high resonators is also neglected because the buoyancy of the side wall is significantly weakened, leading to making conduction predominate over the other heat transfer modes [1-3].

Heat dissipation via natural convection is governed by Newton's law of cooling, which is expressed as follows:

$$q_{\mathbf{conv}} = \bar{h}(T_{\text{ext}} - T), \quad \text{(S4)}$$

where $q_{\mathbf{conv}}$ is the convective heat flux, $\bar{h}$ is the average natural convection coefficient, T_{ext} is the external temperature, and T is the temperature at the surface of the material. The average natural convection coefficients are obtained by two empirical correlations, which are for the cases that natural convection between a hot plate (here, the top surfaces of each resonator and the PDMS) and an upper atmosphere (Eq. S5) and that between a hot plate (here, the bottom surface of the PDMS) and a lower atmosphere (Eq. S6):

$$\bar{h} = \frac{k_{\text{air}}}{L}\left(0.54Ra_L^{1/4}\right), \quad \text{(S5)}$$

$$\bar{h} = \frac{k_{\text{air}}}{L}\left(0.27Ra_L^{1/4}\right), \quad \text{(S6)}$$

where k_{air} is the thermal conductivity of air, L is the characteristic length calculated as the area of the PDMS divided by the perimeter of the PDMS, and Ra_L is the Rayleigh number. The average natural convection coefficients, calculated based on Eq. S5, are 4.73 $\text{W}/m^2 \cdot \text{K}$ for the minimum temperature difference between the external air and the surface of the structure and 11.64 $\text{W}/m^2 \cdot \text{K}$ for the largest temperature difference. The average natural convection coefficients at the bottom of the PDMS film are calculated based on Eq. S6 and are half of those obtained by Eq. S5. Because the natural convection coefficients of gases generally range from 2–25 $\text{W}/m^2 \cdot \text{K}$, the predicted average natural convection coefficients seem to be reasonable [4].

4. Infrared image generation

The three-dimensional (3D) volumetric temperature distribution is acquired through thermal simulation. To transform this 3D distribution into a two-dimensional (2D) infrared image profile, we first calculate the 4D spectral irradiance based on the 3D temperature data.

$$I(x,y,z,\lambda) = \frac{2\epsilon hc^2}{\lambda^5}\frac{1}{\exp\left(\frac{hc}{\lambda k_B T(x,y,z)}\right)-1}, \quad \text{(S7)}$$

where h is the Planck constant, k_B is the Boltzmann constant, c is the speed of light, and ϵ is the emissivity. We assume that the temperature in the surrounding air is at room temperature (298 K). Then the power captured by an infrared camera, with its focal point positioned at the bottom of the substrate, is computed by using a convolution theorem. The axial full-width half-maximum of the point spread function is set as 10 μm. By integrating this spectral irradiance with three dimensions—namely, two spatial dimensions and the wavelength—across the wavelength domain, a 2D heat distribution is derived. Finally, the 2D temperature profile is calculated using the equation $T_{\mathrm{IR}}(x,y) = (Q(x,y)\pi/\sigma\epsilon)^{\frac{1}{4}}$, where π is included for solid angle integration.

5. Robustness of the metasurfaces under fabrication errors

The robustness of the metasurfaces against fabrication errors is presented in this section. Simulations are conducted for three resonator configurations: x/y-aligned resonator (#1), 45°/-45°-aligned resonator (#2), and RCP/LCP resonator (Fig. S2). For the x/y-aligned resonator, total absorption (*A*) and absorption ratio between the two resonators (Q_0/Q_{90}) are obtained under horizontally polarized light at 1 THz by varying L_1 and w_1 (Fig. S2A and B). The formula $\mathrm{L} = L_1 + dL_1$, $\mathrm{w} = w_1 + dw_1$ is used to introduce errors ranging from -10 to 10 μm. These results show that the polarization-sensitive absorption does not degrade significantly upon the fabrication errors. Another noticeable feature is that *A* and Q_0/Q_{90} exhibit the opposite behavior, that is, the parameters resulting in high *A* tends to have lower Q_0/Q_{90}. Our design is optimized to provide high absorption and strong polarization dependence targeted at 1 THz between this trade-off. Indeed, the changes of *A* and Q_0/Q_{90} shown in Fig. S2A and B are associated with the frequency shift. As these results at 1 THz does not show spectral response, the spectra of *A* and Q_0/Q_{90} of the metasurface when *dL1* = 10 μm and *dw1* = -2 μm are presented in Fig. S2C and D. This indicates that the absorption peak is blueshifted. While our original metasurface is focused at 1 THz, it can be tuned for other target frequency by adjusting the geometric

parameters within the same structure.

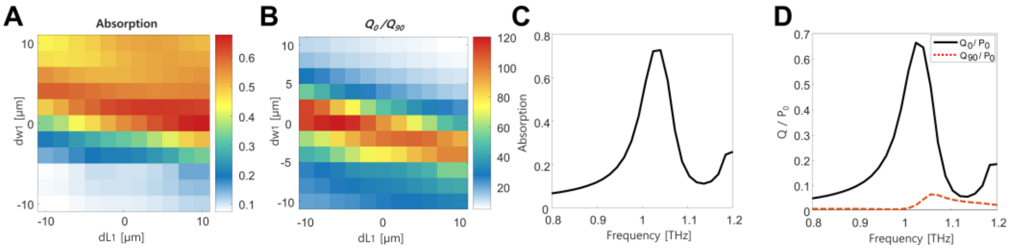

Fig. S2. Optical response of the x/y-aligned resonator changing parameter *L* and *w*. (A) Total absorption and (B) absorption ratio between the two resonators (Q_0/Q_{90}). (C) Total absorption and (D) absorption ratio spectrum when dL_1 = 10 μm and dw_1 = -2 μm.

Similarly, we conduct similar analysis for the 45°/-45°-aligned resonator by changing (L_2, w_2), (L_2, l_2), and (w_2, l_2) (see Fig. S3) and for the RCP/LCP resonator by changing *(a, b)* and θ (see Fig. S4). Overall, the results show the similar tendency with high sensitivity upon the errors in width (*w*), which is natural considering that the absolute length of the width is the smallest and therefore the error of -10 to 10 μm corresponds to the large relative errors. Furthermore, Fig. S4C and D prove that the fabrication error in angle also does not affect the performance significantly.

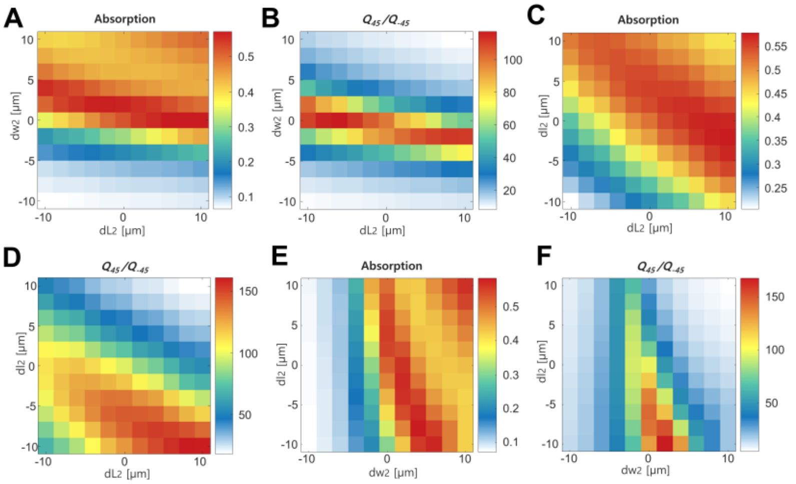

Fig. S3. Optical response of the 45°/-45°-aligned resonator changing parameters (A, B) *L, w* (C, D), *L, l* and (E, F), *w, l*.

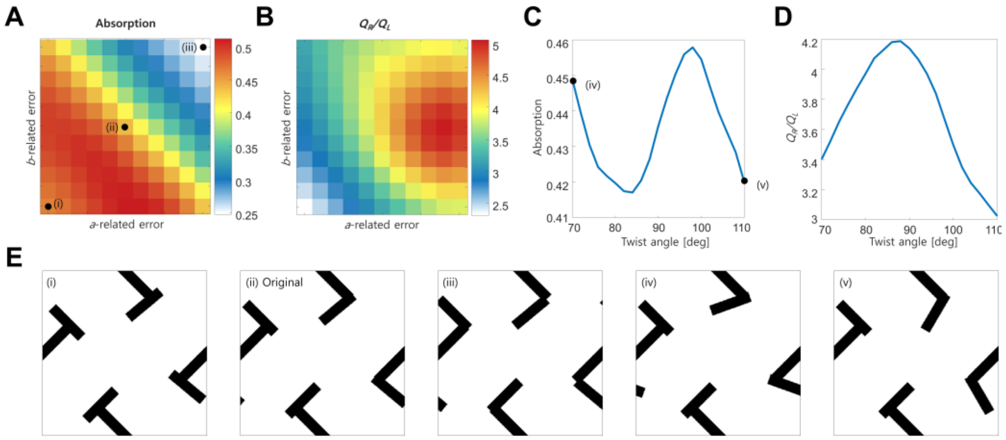

Fig. S4. Optical response of the RCP / LCP resonator changing parameters (A, B) *a, b* and (C, D) θ. For clarification, unit cells at five different points are shown in (E).

6. Steady-state temperature rise under various input powers

In the main manuscript, the input power is 0.1 mW for all thermal simulations. For completeness, we simulate the steady-state temperature of the unit cell consisting of 45° and -45°-aligned resonator under various input power (Fig. S5). When applying 45°-polarized incidence, the two resonators show a distinct temperature rise, indicating that our metasurface enables the polarimetric imaging under different light intensities.

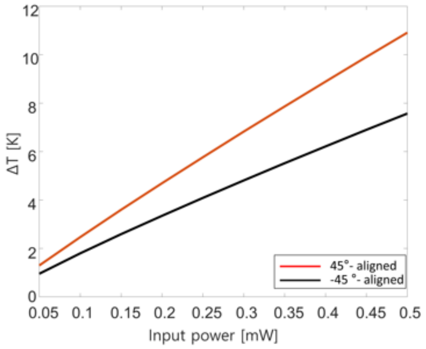

Fig. S5. Steady-state temperature rise of 45° and -45° resonators under various input powers.

7. Thermal response time

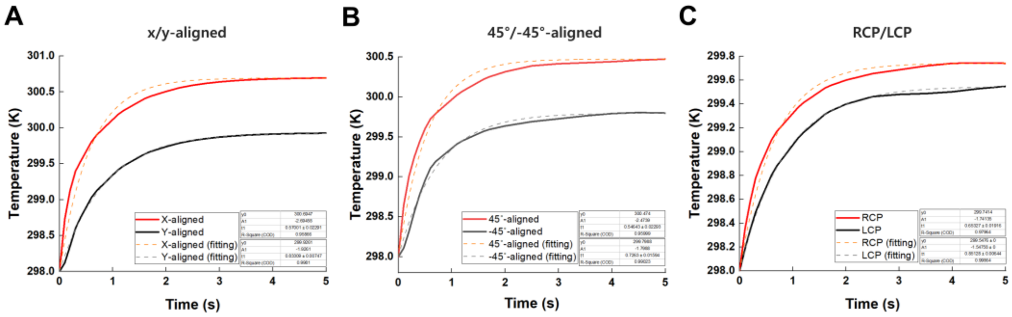

Fig. S6. Fitting of time-transient temperature of unit cells consisting of (A) x/y-aligned, (B) 45°/-45°-aligned, and (C) RCP/LCP resonators.

The thermal response time is conventionally defined as the thermal time constant τ_{th}, indicating that the sensor output signal reaches 63.2% of its the steady-state value [5]. we obtain τ_{th} of each resonator by fitting the temperature profiles shown in Fig. 3 using

$$T(t) = T_i + (T_f - T_i)\left(1 - \exp\left(-\frac{t}{\tau_{th}}\right)\right), \quad \text{(S8)}$$

where T_i and T_f are initial and final temperatures, respectively (Fig. S6). Each resonator approaches the stationary state in different rate and thus has distinct τ_{th}. We define the thermal response time of each resonator as τ_{th}. The thermal response time of each resonator is presented in Table 2.

	Unit cell #1		Unit cell #2		Unit cell #3	
	x-aligned	y-aligned	45°-aligned	-45°-aligned	RCP	LCP
τ_{th} [s]	0.57	0.83	0.55	0.73	0.65	0.85

Table 2. Thermal response time of each resonator.

Therefore, the response times of unit cell #1, #2, and #3 are 0.83, 0.73, and 0.85 seconds, respectively.

References

[1] L. Micheli, K. S. Reddy, and T. K. Mallick, “General correlations among geometry, orientation and thermal performance of natural convective micro-finned heat sinks,” *Int. J. Heat Mass Transf.*, vol. 91, pp. 711–724, Dec. 2015, doi: 10.1016/j.ijheatmasstransfer.2015.08.015.

[2] J. S. Kim, B. K. Park, and J. S. Lee, “NATURAL CONVECTION HEAT TRANSFER AROUND MICROFIN ARRAYS,” *Exp. Heat Transf.*, vol. 21, no. 1, pp. 55–72, Jan. 2008, doi: 10.1080/08916150701647835.

[3] L. Micheli, K. S. Reddy, and T. K. Mallick, “Experimental comparison of micro-scaled plate-fins and pin-fins under natural convection,” *Int. Commun. Heat Mass Transf.*, vol. 75, pp. 59–66, 2016.

[4] T. L. Bergman, *Fundamentals of heat and mass transfer*. John Wiley & Sons, 2011. Accessed: Nov. 09, 2023.

[5] Y. Pan, et al. "Note: Response time characterization of fiber Bragg grating temperature sensor in water medium." Review of Scientific Instruments 87, 11 2016.
